# Assessment of functional capacity and flexibility of patients infected with HTLV-1

**DOI:** 10.1186/1742-4690-12-S1-P39

**Published:** 2015-08-28

**Authors:** Isabelle Rocha Santos, Maria Fernanda Grassi, Ney Boa-Sorte, Ramon de Almeida Kruschewsky, Bernardo Galvão–Castro

**Affiliations:** 1 Centro Integrativo e Interdisciplinar de HTLV, Escola Bahiana de Medicina e Saúde Pública, Salvador, Bahia, Brazil; 2 Laboratório Avançado de Saúde Pública, Centro de Pesquisas Gonçalo Moniz, Fundação Oswaldo Cruz, Salvador, Bahia, Brazil

## 

HTLV-1 infects 5 to 10 million people worldwide. Tropical spastic paraparesis / HTLV associated myelopathy (TSP/HAM) is a neurological disease, that impairs functional capacity, daily life activities and quality of life of infected individual. Objective: To evaluate the functional capacity and flexibility of patients infected with HTLV-1. Methods: Sample was comprised of 65 HTLV-1-infected individuals (41 asymptomatic, 24 TSP/HAM definite) followed at the referral center for HTLV of Bahiana School of Medicine and Public Health in Salvador, Brazil. Twenty-six uninfected individuals were enrolled as controls. Inclusion criteria were age from 18 to 65 years, HTLV-1 serology (ELISA and Western Blot) positive (HTLV-group) and negative (uninfected individuals) and for HTLV-1 infected individuals clinical status defined according to the Belem criteria for the HAM/TSP diagnosis. Patients with stroke or trauma history, those coinfected with HIV, HBV, HCV or syphilis were excluded. The functional capacity of all individuals was assessed using the Group of Latin American development for Maturity (GDLAM) protocol: i) 10 Meter Walking Test, ii) time to get up from a chair, iii) time to get up from the prone position and iv) time to get up from a chair and walk around the house. Test performances were expressed in seconds, and used to calculate the GDLAM index. The flexibility was assessed using the sit and reach test, measured in centimeters. Results: The GDLAM index was significantly lower in the uninfected controls (20.80±3.75 seconds) compared to both asymptomatic (26.45±5.99 seconds) and TSP/HAM patients (51.37±14.99 seconds) (p=0, 1). Moreover, the flexibility of uninfected controls (29.16 ± 7.52cm) was significantly higher compared to asymptomatic HTLV-1-infected individuals (23.36 ± 7.97cm) and HAM/TSP patients (14.45 ± 7.52cm) (p=0, 1). Conclusions: HTLV-1-infeted individuals have an impairment of their functional capacity and flexibility when compared to uninfected individuals, especially patients with TSP/HAM. Interestingly, HTLV-1 asymptomatic individuals also had a decrease of both functional capacity and flexibility, prior to clinically detectable signs of myelopathy.

